# The influence of TAS2R38 bitter taste gene polymorphisms on obesity risk in three racially diverse groups

**DOI:** 10.37796/2211-8039.1175

**Published:** 2021-09-01

**Authors:** Chaowanee Chupeerach, Pradtana Tapanee, Nattira On-Nom, Piya Temviriyanukul, Boonrat Chantong, Nicole Reeder, Grace A. Adegoye, Terezie Tolar-Peterson

**Affiliations:** a Institute of Nutrition, Mahidol University, 999 Phutthamonthon 4 Rd., Salaya, Phutthamonthon, Nakhon Pathom 73170, Thailand; b Department of Preclinical Science and Applied Animal Science, Faculty of Veterinary Science, Mahidol University, 999, Phutthamonthon 4 Rd., Salaya, Phutthamonthon, Nakhon Pathom 73170, Thailand; c Department of Food Science, Nutrition, and Health Promotion, Mississippi State University, MS 39762, USA

**Keywords:** Genetic polymorphism, TAS2R38, Bitter taste receptor, Obesity, Body fat

## Abstract

**Objectives:**

Bitter taste perception affects food preference, eating behavior, and nutrient intake. The purpose of this study was to investigate the contribution of bitter taste gene polymorphisms to body fatness as measured by percentage of body fat.

**Method:**

Three common single nucleotide polymorphisms (SNPs) of the TAS2R38 gene which result in amino acid changes in the protein (A49P, V262A, and I296V), were studied in three racially diverse groups: European Americans n = 313, African Americans n = 109, and Asians n = 234.

**Results:**

The allele frequencies of the three SNPs were similar to previous studies. The rare haplotypes, AAI and AAV, were found in high prevalence in the African American subgroup (22.94%) and European American subgroup (6.07%). The PROP non taster; AVI/AVI diplotype was associated with a higher risk of obesity in European American and Asian but not African American subjects after age adjustment.

**Conclusions:**

TAS2R38 polymorphisms could be associated with obesity development. In addition to taste perception, nutrient sensing and energy metabolism should be studied in relation to bitter taste receptors to confirm the association between genetic polymorphisms and body fatness. Genetic polymorphisms, race, gender, and environmental factors such as dietary patterns could all contribute to body fat.

## 1. Introduction

Humans taste food and other substances by taste receptors that are located in taste buds on the tongue. Taste perception might affect food preference, eating behavior, and nutrient intake, and therefore possibly also contribute to obesity development [1,2]. In humans, bitter taste receptors belong to the class of G-protein coupled receptors (GPCRs) and they are mediated by 25 different gene families; one of them being the *TAS2R38* gene located on chromosome 7q that has been strongly associated with 6-n-propylthiouracil (PROP) sensitivity [3–5]. Three common single nucleotide polymorphisms of *TAS2R38* have been described, all of which result in amino acid changes in the protein alanine to proline at position 49 (A49P), valine to alanine at position 262 (V262A), and isoleucine to valine at position 296 (I296V) [4]. These polymorphisms result in two main haplotypes that are commonly found in the human population: the taster haplotype (PAV), and the non-taster haplotype (AVI) [6]. Other rare haplotypes such as AAV and AAI have been found in the population worldwide [7–9].

Genetic variation in taste sensitivity to PROP has been associated with differences in preference for and selection of bitter tasting fruits and vegetables, sweet tasting foods, added fats, spicy foods, and consumption of alcoholic beverages [2,10–12]. Non-tasters may like a greater variety of foods than tasters; therefore, non-tasters might consume more energy and acquire higher body fatness than tasters [13]. Because taste perception influences eating behavior, taste has been broadly studied as a possible influencing factor for obesity development [14]. In addition to the oral expression of bitter taste receptors for taste sensation, bitter taste receptors are also expressed in other locations such as the human gastrointestinal tract and lungs where they might have a role in nutrient metabolism, the endocrine system, or the immune system [15–17].

It has been shown that PROP tasting and TAS2R38 diplotypes have been associated with perception of other taste stimuli [10,18–21], food preference and choice [22–25] as well as body mass index [26,27]. Additionally, there are associations with metabolic changes with impact on body composition [28–31], anti-oxidant status [31], risk of colonic cancer [32] smoking behavior [33], infections of the respiratory system [34–36], taste impairments [37], attainment of exceptional longevity [38] and even neurode-generative diseases [39–41].

Only a few studies have been conducted to observe the effect of *TAS2R38* genetic polymorphisms in relation to body fatness among diverse ethnicities. Therefore, the present study aimed to investigate the association between *TAS2R38* bitter taste gene polymorphisms and body composition such as body fatness among three ethnic groups.

## 2. Materials and methods

### 2.1. Subjects

This was a cross-sectional study with 656 adults age 18–35 years from three ethnic groups: European Americans, African Americans, and Asians. European American and African American subjects were recruited from Mississippi State University, located in the United States (IRB-17-025), and Asian subjects were recruited from a university located in Thailand (COA NO.2018/072.2903). All subjects provided informed consent prior to participation in the study.

### 2.2. Anthropometric measurements

Height was measured using a calibrated touchless stadiometer (SONARIS; Cardinal/Detecto, Webb City, Mo, USA) in European American and African American subjects and measured using a 235-Heightronic Digital stadiometer in Asian subjects. A bioelectrical impedance analysis scale (TBF-300; Tanita Corp., Tokyo, Japan) was used to estimate body fat percentage and weight in European American and African American subjects, and an Omron body composition monitor (HBF375, Omron Health Care, Tokyo, Japan) was used in Asian subjects. Body fat percentage was classified into categories according to the study of Gallagher, et al. (2000) [42] which for standard adults ages 20–39 years are: underfat (BF%< 20% in females, <10% in males), healthy (BF% 20%–34% in females, 10%–21% in males), and overfat/obese (BF%>34% in females, BF%>21% in males). Body mass index (BMI) values were calculated using the standard BMI equation (kg/m^2^).

### 2.3. Genotyping

Three non-synonymous single nucleotide polymorphisms in *TAS2R38* were genotyped for: rs713598 (A49P), rs1726866 (A262V), and rs10246939 (V296I). TaqMan allelic discrimination assays (ThermoFisher, USA) were used for the study at Mississippi State University, USA. DNA sequencing methods (Macrogen, South Korea) were used for rs1726866 (A262V) and rs10246939 (V296I) and polymerase chain reaction-restriction fragment length polymorphism (PCR-RFLP) with *HaeIII* (Cat. number: ER0151, ThermoFisher, USA) were used for rs713598 (A49P) genotyping in the Asian study. The primers were described elsewhere [11,20]. To ensure accurate recording of TAS2R38 diplotypes from the sequencing method, the professional staff imported all sequences into the BioEdit program (USA) and aligned them to the human reference sequences from NCBI. Second, professional staff used the program to locate and highlight the variations in the aligned sequences. Any samples that showed either a discrepancy between the chromatograph and the computer assignment or a potential rare diplotype were flagged for retesting. Common and rare diplotypes were confirmed in this way.

### 2.4. Statistical analysis

Means and standard deviations were used for quantitative parameters. Frequencies and percentages were used to describe genotype variation among groups. The allele frequencies were calculated to evaluate the differences among populations. Hardy Weinberg equilibrium was also calculated, and statistical analyses were performed using the software GENEPOP [43]. One-way ANOVA with Tukey post-hoc testing was used to describe the differences among genotypes and body composition parameters. Logistic regression was used for odds ratio calculations. P-values < 0.05 were considered statistically significant. The statistical software program SPSS 19.0 for Windows (SPSS Inc., Chicago, IL, USA) was used for analysis.

## 3. Results

### 3.1. Subject characteristics

The characteristics of study participants are reported in Table 1. Six hundred and fifty-six adults with mean age of 21.5 years of age were included in the study. Subjects were comprised of 314 European Americans (48%), 109 African Americans (17%), and 234 Asians (35%). Male subjects represented 15% of European Americans, 17% of African Americans and 30% of Asian participants. BMI and body fat percentage in African Americans were the highest among race groups (Table 1).

### 3.2. TAS2R38 genotype distribution

Genotyping and allelic distributions were analyzed and are described in Table 2. All SNPs were in Hardy-Weinberg Equilibrium with p-values greater than 0.05. The combination of three non-synonymous SNPs caused variation in phenotypes. Eight haplotypes and 20 diplotypes with various frequencies were observed among different races (Table 3). The distribution of *TAS2R38* haplotypes were assessed in study participants. The primary haplotypes observed were PAV (48.78%) and AVI (41.92%), followed by AAI (4.04%) and AAV (3.58%). The rare haplotype AAI had a higher frequency in the African American group (22.94%), and the AAV rare haplotype was found in the European American group (6.07%). These results were similar to previously published studies that described the global diversity in *TAS2R38* [7,9]. Major diplotypes found in the European American group were AVI/ AVI, AVI/PAV, and PAV/PAV; in the Asian group AVI/PAV, PAV/PAV, and AAI/AVI, and in the African American group PAV/AAI (Table 4).

### 3.3. TAS2R38 diplotypes and obesity risk

The association between *TAS2R38* and obesity risk such as body fat percentage (normal versus overfat) was analyzed. To meet the requirements of the statistical test, we excluded diplotypes with a frequency of less than two percent. In this study, gender but not age showed an association with obesity risk (data not shown); therefore, gender was adjusted in the odds ratio calculation for the risk of TAS2R38 diplotypes to obesity. The logistic regression analysis for adjusted odds ratio is shown in Table 5. Interestingly, obesity risk (from the body fat percentage categories) significantly decreased for PAV/PAV (odds ratio; 0.180, 80%) and PAV/AVI (odds ratio; 0.294, 70.6%) when compared to PROP non taster diplotype (AVI/AVI) among the European American group. Similarly for the Asian subjects, PROP taster diplotypes (PAV/PAV, PAV/AVI) were associated with a lower risk for obesity when compared to the PROP non-taster diplotype (AVI/ AVI) with odds ratio; 0.333 (66.7%) and 0.223 (77.7%), respectively. However, no significant associations between TAS2R38 diplotypes and obesity risk were observed in African American subjects.

## 4. Discussion

The present study analyzed *TAS2R38* genetic polymorphisms among European American, African American, and Asian subjects. Three major single nucleotide polymorphisms of the *TAS2R38* gene were genotyped for in the present study. The minor allele types and frequencies differed among races; however, frequencies were similar to those described in previous studies [7,9,11] and in the dbSNP database (NCBI). Race was an important variable because rare haplotypes were observed in some racial groups. In European American and African American subjects, rare haplotypes including AAV and AAI were found, respectively. The present study showed similar results to a previous study [8], in which the African American group had greater genetic diversity in *TAS2R38* than other races. Thus, the role of bitter taste gene polymorphisms and bitter taste sensitivity to health status is more complex in the African American population than previously known. This could be because the African American population is genetically a very diverse group. However, the Asian population had the lowest prevalence of rare haplotypes in *TAS2R3*8 that similar with previous studies [44,45].

As was hypothesized in previous studies, bitter taste gene polymorphisms might affect taste perception, alter eating behavior, and may be a contributing factor for the development of obesity or other metabolic diseases [2,10–12,26–31]. The PAV and AVI haplotypes of *TAS2R38* represent the PROP taster and non-taster phenotypes, respectively. Other haplotypes including rare haplotypes have been reported as intermediate or supertaster phenotypes [7,20,46]. As was observed in the present study, the homozygous AVI/AVI diplotype (non-taster diplotype), was associated with a higher risk for obesity than the heterozygous AVI/PAV and homozygous PAV/PAV diplotypes in European American and Asian subjects after adjustment for age. This might be explained by previous studies that reported PROP non-tasters have reported to have higher reported food intakes, higher intake of fruit, vegetables, and spicy food, and also higher alcohol consumption [13,22–25,33,46]. PROP tasters might be sensitive to foods with a bitter aftertaste, sweet and fatty foods, spicy foods, and have scored higher overall on food disliking [47,48]. PAV allele carrier might potentially demonstrate more selective eating behavior pattern [49]. In this study, there was no association between non taster and other rare diplotypes. Few studies have reported on associations between common and rare haplotypes with obesity markers such as body fat percentage or BMI because they did not have large enough frequencies for statistical analysis [49,50]. Moreover, body fat percentage was used as obesity risk factor instead of body mass index which could make the study difference or more accurate [42,52]. However, it is difficult to conclude the effect of these genetic polymorphisms on obesity development among African American subjects.

*TAS2R38* haplotype predicts the majority (55–85%) of phenotypic variance in PROP sensitivity [51–54]. These genetic polymorphisms might regulate the receptor function which differs from taste perception function. Bitter taste receptors are G protein coupled receptors, and in addition to being present in taste buds, are also present in the gastrointestinal system, respiratory system, and brain, although their function in these locations is not well understood [53–56]. Taste receptors could also function in nutrient sensing [17], endocrine regulation, or energy metabolism such glucose homeostasis [1,15,30]. The expression of *TAS2R38* in the intestines might influence energy balance and intraluminal changes occurring in obesity [17,57]. Moreover, three candidate genetic polymorphism could change the gut TAS2R38 receptor by the alteration of ligand binding site. These data suggested that the extra oral of TAS2R38 could modulate the release of gut peptides and possibly caused in metabolic effects [58]. Therefore, the genetic polymorphism of TAS2R38 might associate with obesity risk as in this present study.

We identified several limitations to our study. We did not measure PROP sensitivity in study participants therefore we lack the association between genotype and taste perception phenotype. We did not measure other bitter and non-bitter taste gene polymorphisms which could be functioning together with *TAS2R38* [59]. Other forms of bitter taste or other taste receptors might be involved in metabolic control, resulting in changes in obesity risk [21,28,29]. Taste bud density was not measured, but it may also affect taste perception. Moreover, the present study did not compare genetic polymorphisms in Native Americans which might differ in the gene pattern from European or African Americans [60]. The influence of exposure to different lifestyle factors among subject groups might also be associated with differences in obesity risk. Moreover, the use of bioelectrical impedance analysis for evaluation of fat mass and the effect of population stratification in admixed populations should be considered in future studies to confirm the contribution of TAS2R38 SNPs to the risk of developing obesity.

## 5. Conclusion

The PROP non-taster diplotype was associated with a higher risk for obesity in European Americans and Asians after adjustment for sex. These diplotypes relate to bitter taste perception and to eating behavior which affects food choice but not energy intake, or they might affect extra-oral functions such as gastrointestinal function. The mechanisms could not be clarified in the present study. Race, sex, and environmental factors cannot be excluded from the complex relationship between genetic polymorphisms and obesity.

## Figures and Tables

**Table 1:** Descriptive characteristics of study participants.

Characteristic	European-American (N = 314)	African-American (N = 109)	Asian (N = 234)
Mean age (years)	20.2 ± 2.4^a^	20.0 ± 1.5^a^	24.2 ± 7.0^b^
Sex (N, %)			
Male	48 (15.3)	18 (16.5)	69 (29.5)
Female	266 (84.7)	91 (83.5)	165 (70.5)
BMI	23.79 ± 4.83^a^	26.26 ± 7.32^b^	22.94 ± 4.73^a^
Fat%	25.99 ± 8.54^a^	29.66 ± 11.29^b^	25.07 ± 7.25^a^
Body fat % category			
Underfat	52 (16.6)	14 (12.8)	67 (28.8)
Normal fat	200 (63.9)	51 (46.8)	128 (54.9)
Overfat	61 (19.5)	44 (40.4)	38 (16.3)

Different letters show statistical significance among race groups. ANOVA with Tukey post-hoc tests were used and p values less than 0.05 were considered statistically significant. Body fat % categories for adults ages 20–39 years are underfat (BF%< 20% in females, <10% in males), healthy (BF% 20%–34% in females, 10%–21% in males), and overfat/obese (BF%>34% in females, BF% > 21% in males).

**Table 2:** Genotype and allele distribution of TAS2R38 in the study group.

SNP	Race	Genotype N (%)			MAF	P value
	
rs713598		AA	AP	PP		
	European American	109 (34.7)	147 (46.8)	58 (18.5)	P = 0.42	0.233
	African American	34 (31.2)	57 (52.3)	18 (16.5)	P = 0.43	0.800
	Asian	31 (13.2)	103 (44.0)	100 (42.7)	A = 0.35	0.337

rs1726866		VV	VA	AA		
				
	European American	88 (28.0)	151 (48.1)	75 (23.9)	A = 0.48	0.317
	African American	13 (11.9)	50 (45.9)	46 (42.2)	V = 0.35	0.596
	Asian	34 (14.5)	98 (41.9)	102 (43.6)	V = 0.35	0.116

rs10246939		II	IV	VV		
				
	European American	75 (23.9)	149 (47.5)	90 (28.7)	I = 0.48	0.226
	African American	34 (31.2)	57 (52.3)	18 (16.5)	V = 0.43	0.813
	Asian	35 (15.0)	97 (41.5)	102 (43.6)	I = 0.36	0.087

MAF; minor allele frequency, P value for Hardy Weinberg Equilibrium, Homozygous allele; AA (alanine), VV (valine), II (isoleucine), PP (proline), Heterozygous allele; AP, VA, IV.

**Table 3:** Haplotype percentage distributions of TAS2R38 in the study group.

Population	PAV	AVI	AAV	AVV	PAI	PVI	AAI	PVV
Total	48.78	41.92	3.58	0.23	0.30	0.91	4.04	0.23
European-American	40.73	51.12	6.07	0.48	0.32	0.48	0.48	0.32
African-American	42.20	33.94	0.92	0.00	0.00	0.00	22.94	0.00
Asian	62.61	33.33	1.50	0.00	0.43	1.92	0.00	0.21

Haplotype: subset of allele from multiple loci on a single chromosome.

**Table 4:** Distribution of TAS2R38 diplotype by race.

Diplotype	European American	African American	Asian
AVI/AVI	85 (27.16)	12 (11.01)	30 (12.82)
AVI/PAV	128 (40.89)	33 (30.28)	91 (38.89)
PAV/PAV	55 (17.57)	18 (16.51)	95 (40.60)
AAV/AAV	2 (0.64)	-	-
PVI/PVI	1 (0.32)	-	1 (0.43)
AAV/AVI	18 (5.75)	2 (1.83)	-
AVV/AVV	1 (0.32)	-	-
PAV/AAV	15 (4.79)	-	7 (2.99)
PAV/AVV	1 (0.32)	-	-
PAI/PAI	1 (0.32)	-	-
AAV/AAI	1 (0.32)	-	-
PVI/AVI	1 (0.32)	-	2 (0.85)
PAV/PVV	1 (0.32)	-	-
AAI/AVI	2 (0.64)	14 (12.84)	-
PVV/AVI	1 (0.32)		1 (0.43)
PAV/AAI	-	22 (20.18)	-
PAV/AVI	-	1 (0.92)	-
AAI/AAI	-	7 (6.42)	-
PAV/PVI	-	-	2.14
AVI/PAI	-	-	0.85

Diplotypes; a variant of all possible combined haplotypes in a population.

**Table 5:** The risk of TAS2R38 diplotype to obesity in each race group.

Parameters	Number (%)		Adjusted Odds ratio	95% CI	P value

	Normal	Obese			
European American					
Gender					
Female	164 (79)	44 (21)			
Male	29 (67)	14 (33)	1.294	0.619–2.704	0.494
Diplotype					
AVI/AVI (reference)	46 (72)	18 (28)	-	-	-
PAV/PAV	37 (84)	7 (16)	0.180	0.079–0.410	<0.001
PAV/AVI	87 (76)	27 (23)	0.294	0.185–0.467	<0.001
AAV/AVI	9 (60)	6 (40)	0.611	0.211–1.773	0.365
PAV/AAV	14 (100)	0 (0)	-	-	-

African American					
Gender					
Male	8 (17)	38 (83)			
Female	38 (53)	34 (47)	0.612	0.186–2.006	0.417
Diplotype					
AVI/AVI (reference)	7 (70)	3 (30)	-	-	-
PAV/AVI	18 (67)	9 (33)	0.526	0.234–1.181	0.120
PAV/PAV	8 (50)	8 (50)	1.063	0.393–2.869	0.905
PAV/AAI	8 (40)	12 (60)	1.618	0.647–4.050	0.304
AAI/AVI	5 (42)	7 (58)	1.460	0.460–4.633	0.521

Asian					
Gender					
Female	86 (83)	17 (17)			
Male	33 (66)	17 (34)	1.454	0.711–2.974	0.305
Diplotype					
AVI/AVI (reference)	20 (87)	3 (13)	-	-	-
PAV/AVI	51 (80)	13 (20)	0.223	0.115–0.435	<0.001
PAV/PAV	48 (73)	18 (27)	0.333	0.183–0.603	<0.001

Normal and obese criteria characterized by normal and overfat cut-off. Under-fat was excluded from this analysis. Logistic regression analyzed with Enter method. P value less than 0.05 was statistical significant.
